# Effects of 3,4-Methylenedioxymethamphetamine Administration on Retinal Physiology in the Rat

**DOI:** 10.1371/journal.pone.0029583

**Published:** 2011-12-27

**Authors:** João Martins, Miguel Castelo-Branco, Ana Batista, Bárbara Oliveiros, Ana Raquel Santiago, Joana Galvão, Eduarda Fernandes, Félix Carvalho, Cláudia Cavadas, António F. Ambrósio

**Affiliations:** 1 Centre of Ophthalmology and Vision Sciences, IBILI, Faculty of Medicine, University of Coimbra, Coimbra, Portugal; 2 Centre for Neuroscience and Cell Biology (CNC), University of Coimbra, Coimbra, Portugal; 3 REQUIMTE - Laboratory of Toxicology, Department of Biological Sciences, Faculty of Pharmacy, University of Porto, Porto, Portugal; 4 REQUIMTE - Department of Chemical Sciences, Faculty of Pharmacy, University of Porto, Porto, Portugal; 5 Faculty of Pharmacy, University of Coimbra, Coimbra, Portugal; 6 AIBILI, Coimbra, Portugal; Institut de la Vision, France

## Abstract

3,4-Methylenedioxymethamphetamine (MDMA; ecstasy) is known to produce euphoric states, but may also cause adverse consequences in humans, such as hyperthermia and neurocognitive deficits. Although MDMA consumption has been associated with visual problems, the effects of this recreational drug in retinal physiology have not been addressed hitherto. In this work, we evaluated the effect of a single MDMA administration in the rat electroretinogram (ERG). Wistar rats were administered MDMA (15 mg/kg) or saline and ERGs were recorded before (Baseline ERG), and 3 h, 24 h, and 7 days after treatment. A high temperature (HT) saline-treated control group was also included. Overall, significantly augmented and shorter latency ERG responses were found in MDMA and HT groups 3 h after treatment when compared to Baseline. Twenty-four hours after treatment some of the alterations found at 3 h, mainly characterized by shorter latency, tended to return to Baseline values. However, MDMA-treated animals still presented increased scotopic a-wave and b-wave amplitudes compared to Baseline ERGs, which were independent of temperature elevation though the latter might underlie the acute ERG alterations observed 3 h after MDMA administration. Seven days after MDMA administration recovery from these effects had occurred. The effects seem to stem from specific changes observed at the a-wave level, which indicates that MDMA affects subacutely (at 24 h) retinal physiology at the outer retinal (photoreceptor/bipolar) layers. In conclusion, we have found direct evidence that MDMA causes subacute enhancement of the outer retinal responses (most prominent in the a-wave), though ERG alterations resume within one week. These changes in photoreceptor/bipolar cell physiology may have implications for the understanding of the subacute visual manifestations induced by MDMA in humans.

## Introduction

3,4-methylenedioxymethamphetamine (MDMA; ecstasy) is a recreational drug often consumed at “rave” parties. It is a serotonergic agent with neurotoxic potential [1], [2]. Although the death rate caused by ecstasy in humans is relatively low compared to other drugs of abuse, it has been associated with potential fatal consequences, including hyperthermia, rhabdomyolysis, hepatotoxicity, cardiotoxicity, and acute renal failure [3], [4], [5]. It has been also associated with significant deficits in neurocognitive function, mainly verbal memory [1], [6].

Characteristic psychotropic effects are thought to be caused by enhanced serotonin (5-hydroxytryptamine, 5-HT) release, although some effects may also involve norepinephrine and dopamine (DA) [1], [7]. In laboratory animals, MDMA administration is known to induce an acute and rapid release of both 5-HT [8] and DA [9] from serotonergic and dopaminergic nerve endings. The acute release can be followed by long-term neurotoxic transmitter depletion, mainly 5-HT, in some brain regions, as experimentally observed in the rat [10], [11] and monkey [12]. However, it is not yet clear whether these MDMA neurotoxic effects underlie overt cognitive deficits. This is still a matter of controversy and further research is needed [13].

MDMA induces programmed cell death, with a significant increase of apoptosis markers in the amygdala and hippocampus [14], and also in cerebellar granule [15] and cortical [16] neuronal cultures. Unlike the numerous available brain research reports, there are scarce studies addressing the effects of MDMA in the retina and vision. Changes in light perception were demonstrated in humans after the administration of repeated doses of MDMA [17], [18], [19]. Moreover, the drug has been found in human vitreous humor after MDMA ingestion [20], [21], and vitreal MDMA quantification was proposed as a reliable way to measure the MDMA concentration for toxicological analysis [22]. Also, there are a few clinical case reports of ocular problems after MDMA ingestion [23], [24]. There is only one study evaluating the effect of MDMA administration in the rat retina [25]. The authors found that the number of TUNEL-positive nuclei increased significantly in rat retinas after one week of daily MDMA administration. We also showed that exposure of cultured retinal neural cells to MDMA induces cell degeneration [26]. Taken together, these studies suggest that MDMA can affect retinal physiology in addition to its harmful effects to the brain.

Taking the scarcity of direct physiological studies into account, we evaluated potential alterations in the rat retinal light responses induced by a single peripheral administration of MDMA. We used electroretinography, which is a technique that measures global retinal population responses which tend to be dominated by particular groups of retinal layers allowing pathophysiological interpretation [27], [28], [29]. We found MDMA-induced ERG changes that were independent of elevated temperature, in particular for the outer retinal layers. The MDMA-induced effects were present 24 h after drug administration though they resume after 7 days. These findings suggest a direct retinal effect of MDMA that may have implications for the understanding of visual perceptual effects induced by this drug.

## Methods

### Animals and drug administration

All procedures involving animals were in accordance with the Association for Research in Vision and Ophthalmology (ARVO) statement for vision and ophthalmic research, and were approved by our Institutional Ethics Committee (*Comissão de Ética da Faculdade de Medicina da Universidade de Coimbra*). Approval ID: FMUC/12/09. Ethics Committee for Animal Experiments: Manuel Amaro de Matos Santos Rosa, MD/PhD; Paulo de Carvalho Pereira, PhD; António Francisco Rosa Gomes Ambrósio, PhD; Flávio Nelson Fernandes Reis, PhD (Ethics Statement). Male Wistar rats aged 8 weeks were maintained at 22±1°C on a 12 h light/12 h dark cycle, with access to water and food *ad libitum*. In the first study, 30 animals were divided into three groups: Control (n = 10), high temperature (HT, n = 8), and MDMA (n = 12). In a second study (7 days ERGs) 24 animals were divided into two groups: Control (n = 12) and MDMA (n = 12). The rationale for the high temperature group is to mimic the temperature elevation detected in MDMA-treated animals, since body temperature affects ERG recordings [30]. MDMA-treated animals received one single intraperitoneal (i.p.) injection of racemic MDMA (15 mg/kg). Control and HT animals received one single i.p. injection of saline (0.9% NaCl). I.p. injections were performed under dim red light.

### ERG recordings

ERGs were recorded after overnight dark adaptation. In the first study, ERGs were recorded 24 h before treatment (i.p. injection), 3 h after treatment; and 24 h after treatment. In the second study, ERGs were recorded 7 days before, and 7 days after treatment (i.p. injection). In both studies, the ERGs performed before treatment were considered Baseline ERGs. The animals were anesthetized by intramuscular injection of ketamine hydrochloride (50 mg/kg) and xylazine (10 mg/kg) and the pupil fully dilated with topical tropicamide (1%) under dim red light illumination. The body temperature was maintained with a heating pad set to 37°C during the procedure, except for the high temperature animals at the 3 h time point in the first study. In that case, the heating pad was set to 45°C during 10 min before ERG recording to induce an elevation in the animal body temperature (37.6±0.5°C) to mimic the temperature found in animals administered MDMA at the same time point (37.4±0.6°C in the MDMA group compared to 35.3±0.8°C in the Control group). Using a Ganzfeld stimulator, series of white light flashes ranging from 0.0095 to 9.49 cd-s/m^2^ were applied under scotopic and photopic conditions (in the latter case, after light adaptation to a white background (25 cd/m^2^). A photopic flicker test was also performed where white bright flashes (3.00 and 9.49 cd-s/m^2^) were delivered ten times at 6.3 Hz. ERGs were recorded with a corneal gold wire electrode, a reference electrode at the head, and a ground electrode in the tail. A band width of 1–300 Hz and sampling rate of 3.4 kHz (0.65 kHz for flicker test) were used for acquisition (Roland Consult GmbH, Brandenburg, Germany). OFF-line digital filters were applied on the b-wave (high frequency cut-off of 50 Hz) and oscillatory potentials (low frequency cut-off of 60 Hz for scotopic ERGs and 55 Hz for photopic ERGs) with the RETIport software (Roland Consult GmbH, Brandenburg, Germany).

### Statistical analysis

These experiments were designed to compare temporal effects across treatments within subjects. We have therefore applied, in the first study, mixed between-within subjects (repeated measures) ANOVA. Effects were tested along time (Baseline, 3 h, 24 h) as within-subject factors, and the type of pharmacological manipulation (Control, HT, MDMA) was the between-subject (group) factor. An additional factor was the interaction term between time and group (Wilks' lambda test). This analysis was followed by post hoc paired *t* test. In the second study paired *t* tests were used to compare temporal effects across treatments within subjects. *P* values at a stringent criterion of less than 0.01 were taken as significant. In figures, temporal data are normalized to Baseline to better emphasize within subject effects. All values are presented as mean ± SEM.

## Results

To evaluate the potential effects of MDMA on retinal physiology, scotopic and photopic ERGs were recorded in animals administered with MDMA (15 mg/kg; single administration). Since MDMA induces acute hyperthermia, which affects ERG responses, we included a group of animals (saline-treated) with increased body temperature (high temperature; HT) only at 3 h time point, in the first study. In the second study, only Control and MDMA groups were included in order to evaluate the effect of MDMA (15 mg/kg; single administration) 7 days after i.p. injection.

### Differential effects of MDMA and HT on ERG responses under scotopic conditions

#### a-wave and b-wave after 3 h and 24 h

**Figure 1:**
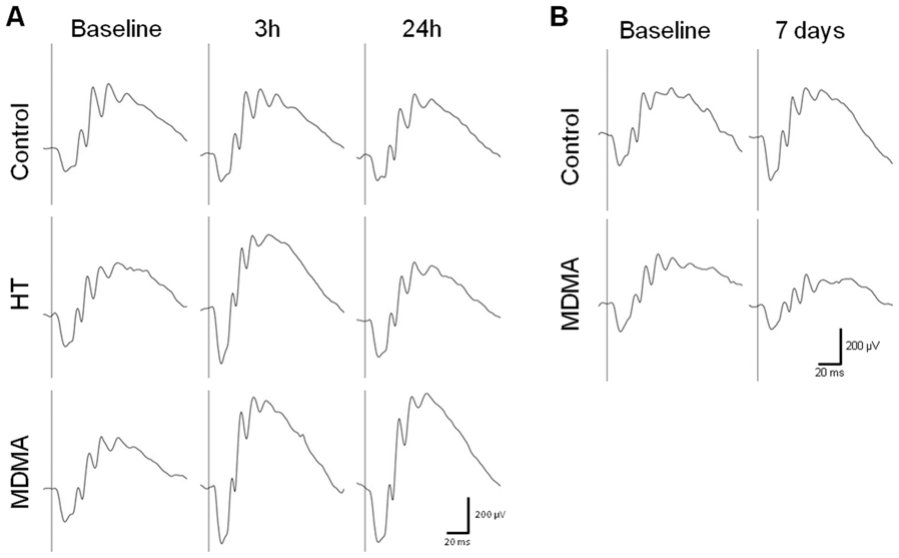
Effect of MDMA administration and high temperature (HT) on scotopic ERGs. Representative traces of individual scotopic ERGs recorded 24 h before saline or MDMA i.p. injection (Baseline ERG), and 3 h and 24 h after i.p. injection (A), and also recorded 7 days before (Baseline ERG) and 7 days after i.p. injection (B) . Waveforms were elicited by flash light stimuli with 3.0 cd-s/m^2^. Each trace represents an average of three responses. Solid vertical lines indicate the onset of flash light stimuli.

ERGs were recorded 24 h before (Baseline ERG), and 3 h and 24 h after saline or MDMA i.p. injection (Fig. 1A), in the first study. In the second study, ERGs were performed 7 days before (Baseline ERG) and 7 days after i.p. injection (Fig. 1B). Waveforms were elicited by flash light stimuli with intensities ranging from 0.0095 to 9.49 cd-s/m^2^.

**Figure 2:**
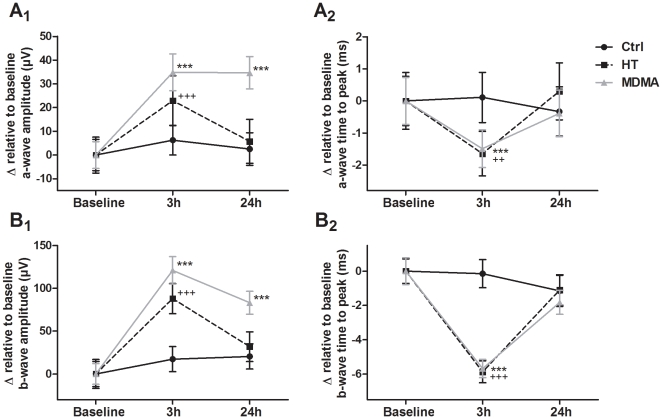
Effect of MDMA and HT on the a-wave and b-wave at 3 h and 24 h. The a-wave (A) and b-wave (B) were extracted from scotopic ERG recordings. The mean values of each Baseline ERG analyzed were subtracted to 3 h and 24 h ERG values in the same group and the obtained delta values (Δ) are presented for graphical illustration. (A_1_,B_1_) Both MDMA and HT groups had increased a-wave and b-wave amplitudes at 3 h time point, while no differences were found in the Control group when responses were compared to the Baseline ERG. At 24 h time point only MDMA group presented increased a-wave and b-wave amplitude values when compared to Baseline ERG. (A_2_, B_2_) Both MDMA and HT groups presented decreased time to peak values at 3 h time point when compared to Baseline ERG in both the a-wave and b-wave, while no significant differences were found at 24 h time point compared to Baseline ERG in all groups. *** p<0.001 MDMA group compared to Baseline ERG; ++ p<0.01, +++ p<0.001 HT group compared to Baseline ERG. The data are presented as mean ± SEM.

The a-wave values were extracted from scotopic ERG recordings elicited by six different light intensities (0.03 – 9.49 cd-s/m^2^) since at 0.00095 cd-s/m^2^ the a-wave was not detected. In the first study, the ANOVA within subject analysis indicated a significant main effect of time for amplitude (F_(2,143)_ = 38.6; p<0.001) (Fig. 2A_1_) and time to peak (F_(2,143)_ = 26.8; p<0.001) (Fig. 2A_2_). There was also an interaction between time and group in both amplitude and time to peak values (p<0.001). Post hoc analyses showed that both MDMA-treated and HT groups had increased a-wave amplitudes (p<0.001) and decreased time to peak values (p<0.001 for MDMA and p<0.01 for HT groups) at the 3 h time point, when compared to Baseline ERG, while the Control group showed no differences. This result suggests an increased and “accelerated” (fast onset) photic response of photoreceptors after MDMA administration, mainly due to body temperature elevation, since HT group had a similar behavior. Importantly, at the 24 h time point, only the MDMA-treated group showed increased amplitude values when compared to Baseline ERG (p<0.001), while in both Control and HT groups the a-wave amplitude values returned to Baseline levels (Fig 2A_1_). This shows that a factor other than temperature might be associated with changes induced by MDMA in the a-wave 24 h after drug administration. Time to peak values at 24 h time point showed no difference when compared to Baseline ERG for all groups.

The b-wave values were extracted from scotopic ERG recordings elicited by seven different light intensities (0.0095–9.49 cd-s/m^2^). The ANOVA repeated measures (within subjects) analysis indicated a main effect of time for amplitude (F_(2,167)_ = 62.3; p<0.001) (Fig. 2B_1_) and time to peak (F_(2,167)_ = 29.2; p<0.001) (Fig. 2B_2_), and a significant interaction between time and group for both amplitude and time to peak values (p<0.001). Similarly to the a-wave, results of the post hoc analysis for the b-wave showed that both MDMA and HT groups had increased amplitudes and decreased time to peak values at the 3 h time point when compared to Baseline ERG (p<0.001), while in Control group no differences were observed. Remarkably, and as observed for the a-wave, 24 h after treatment, only the MDMA group presented increased amplitude values when compared to Baseline (p<0.001), while in both Control and HT groups the b-wave amplitude remained unchanged (Fig 2B_1_). Time to peak values at 24 h time point presented no differences when compared to Baseline ERG for all groups (Fig 2B_2_). Together these results suggest that temperature elevation causes the augmented and “accelerated” (fast onset) b-wave 3 h after MDMA administration, since a similar behavior was detected in the HT group. However, and in agreement with the results obtained concerning the a-wave, they show that a factor other than temperature is implicated in MDMA-induced increases in the b-wave amplitude observed 24 h after MDMA administration.

Regarding the b/a amplitude ratio, ANOVA mixed between and within subjects analysis indicated that there were no significant effects (data not shown), suggesting that the effects observed at the b-wave level result from alterations at the a-wave level (no amplification in effect size).

#### a-wave and b-wave after 7 days

**Figure 3:**
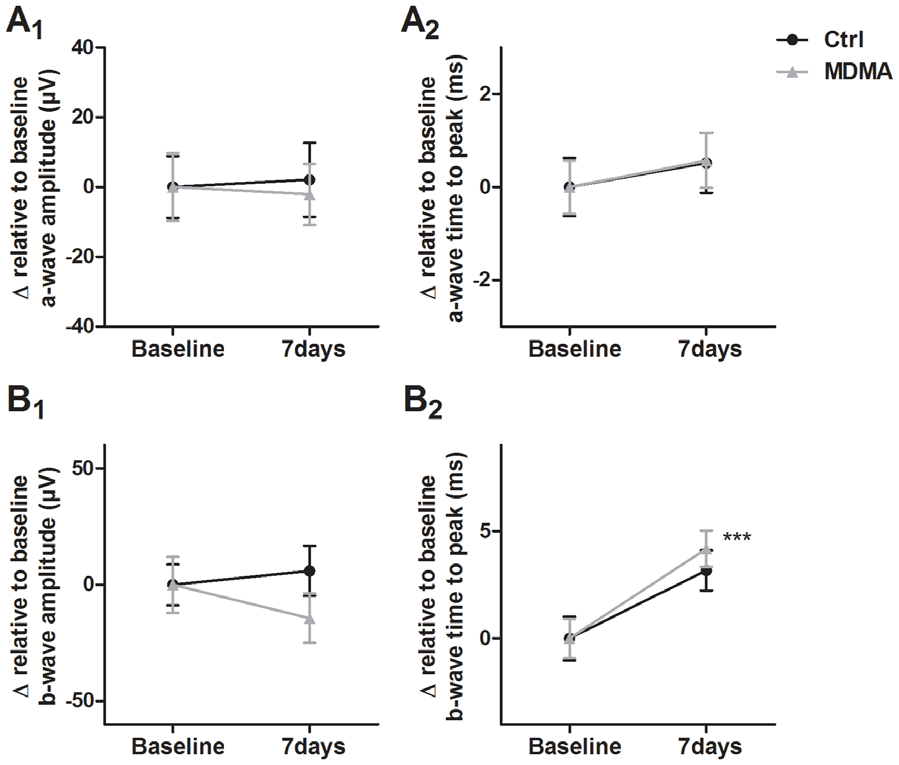
Effect of MDMA on the a-wave and b-wave at 7 days. The a-wave (A) and b-wave (B) were extracted from scotopic ERG recordings. The mean values of each Baseline ERG analyzed were subtracted to 7 days ERG values in the same group and the obtained delta values (Δ) are presented for graphical illustration. (A_1_, B_1_) No differences were found both in the Control and MDMA group in the a-wave and b-wave amplitudes 7 days after i.p. injection when compared to Baseline ERG. (A_2_) No differences were found both in Control and MDMA groups in the a-wave time to peak values. (B_2_) At 7 days, MDMA group presented increased time to peak values in the b-wave, when compared to Baseline ERG, though the Control group show a similar trend (p = 0.015). *** p<0.001 MDMA group compared to Baseline ERG. The data are presented as mean ± SEM.

In the second study, ERGs were recorded 7 days before (Baseline ERG) and 7 days after i.p. injection. The a-wave (Fig 3A) and b-wave (Fig 3B) amplitude and time to peak values were extracted as in the first study. The paired-samples *t* test showed that there were no differences in the a-wave both in Control and MDMA groups at 7 days when compared to Baseline ERG, either in the a-wave amplitude (Fig 3A_1_) or in the a-wave time to peak values (Fig 3A_2_). The b-wave amplitude showed similar behavior to the a-wave with no significant differences in both Control and MDMA groups (Fig 3B_1_). However, regarding time to peak, the MDMA group presented increased values at 7 days when compared to Baseline ERG (p<0.001), though the Control group showed a similar trend (Fig 3B_2_). These results suggest that the augmented amplitudes of the a-wave and b-wave induced by MDMA administration at 24 h were not present 7 days after i.p. injection.

#### Oscillatory potentials at 3 h and 24 h

**Figure 4:**
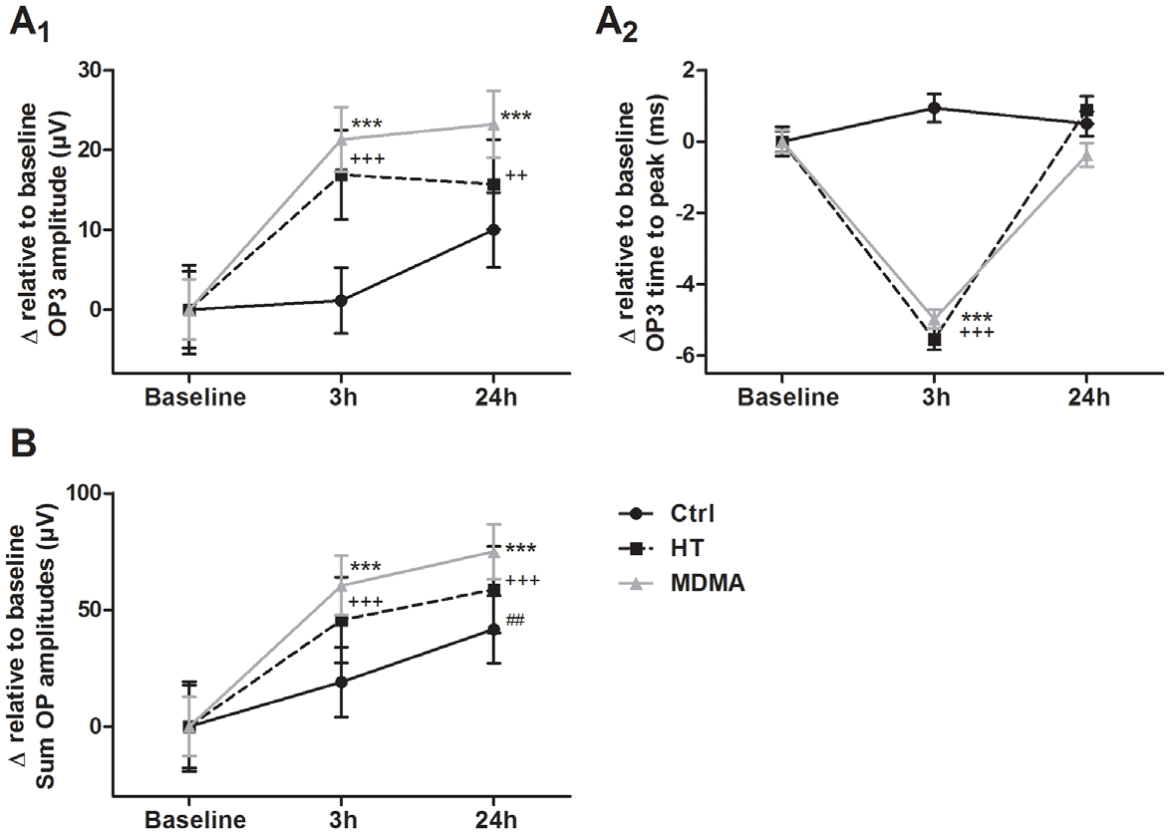
Effect of MDMA and HT on the scotopic OPs at 3 h and 24 h. Four oscillatory potentials (OPs), OP1, OP2, OP3, and OP4, were extracted from scotopic ERGs. (A) Only the effect on the OP3 amplitude and time to peak is shown. (A_1_) Both MDMA and HT groups had increased OP3 amplitudes at 3 h and 24 h time points when compared to Baseline ERG, while no differences were found in the Control group. (A_2_) Time to peak values showed the same behavior for all OPs, in which both MDMA and HT groups presented decreased time to peak values at 3 h time point when compared to Baseline ERG. At 24 h time point similar values were obtained in all groups. (B) Both MDMA and HT groups had increased Sum of OP amplitudes at 3 h and 24 h time points when compared to Baseline ERG. However, at 24 h time point, the Control group also presented increased amplitudes when compared to Baseline ERG. *** p<0.001 MDMA group compared to Baseline ERG; ++ p<0.01, +++ p<0.001 HT group compared to Baseline ERG; ## p<0.01 Control group compared to Baseline ERG. The data are presented as mean ± SEM.

Four oscillatory potential waveforms (OPs), OP1, OP2, OP3, and OP4, were extracted from scotopic ERGs elicited by five different light intensities (0.095 – 9.49 cd-s/m^2^). For the OP amplitudes the ANOVA repeated measures (within subjects) analysis indicated that although there is a main effect of time for all OPs: OP1 (F_(2,118)_ = 45.4; p<0.001); OP2 (F_(2,119)_ = 32.4; p<0.001); OP3 (F_(2,119)_ = 35.5; p<0.001); and OP4 (F_(2,119)_ = 13.8; p<0.001), there is a significant interaction between time and group only for the OP3 (p<0.001) (Fig. 4A_1_), and OP4 (p<0.001) (data shown only for the OP3 component), though OP2 showed a marginally significant interaction effect (p = 0.047). Post hoc analysis showed that both MDMA and HT groups presented increased OP3 amplitude at 3 h (p<0.001) and 24 h (p<0.001 for MDMA and p<0.01 for HT) time points when compared to Baseline ERG (Fig 4A_1_). This result suggests the existence of a prolonged effect caused by elevated temperature in MDMA-induced OP amplification 3 h after drug administration. Contrarily to the observations concerning the a-wave and b-wave, in this particular case, at 24 h time point, it was not possible to discard a temperature contribution since the HT animals also showed increased amplitudes when compared to Baseline ERG. The results obtained for the Sum of OP amplitudes corroborate the hypothesis that no genuine effect of MDMA is found in this parameter since, at 24 h time point, all groups presented increased values when compared to Baseline ERG (p<0.001 for MDMA and HT groups; p<0.01 for Control group; Fig 4B).

Time to peak values followed the pattern observed for the a-wave and b-wave (Fig. 4A_2_). ANOVA repeated measures analysis indicated a main effect of time for all OPs: OP1 (F_(2,118)_ = 148; p<0.001); OP2 (F_(2,119)_ = 170; p<0.001); OP3 (F_(2,119)_ = 142; p<0.001); and OP4 (F_(2,119)_ = 150; p<0.001). There was also a significant interaction between time and group, for OP1, OP2, OP3, and OP4 (p<0.001). Post hoc analysis confirmed a similar behavior as for the a-wave and b-wave, in which both MDMA and HT groups presented decreased time to peak values at 3 h time point (p<0.001), while in the Control group no changes were detected. In addition, no differences were found for all OPs 24 h after treatment, when compared to Baseline ERG (Fig 4A_2_). This result confirms that the temperature elevation is implicated in the “accelerated” (fast onset) response found in MDMA-treated animals, as the behavior was identical to that found in HT animals.

#### Oscillatory potentials at 7 days

**Figure 5:**
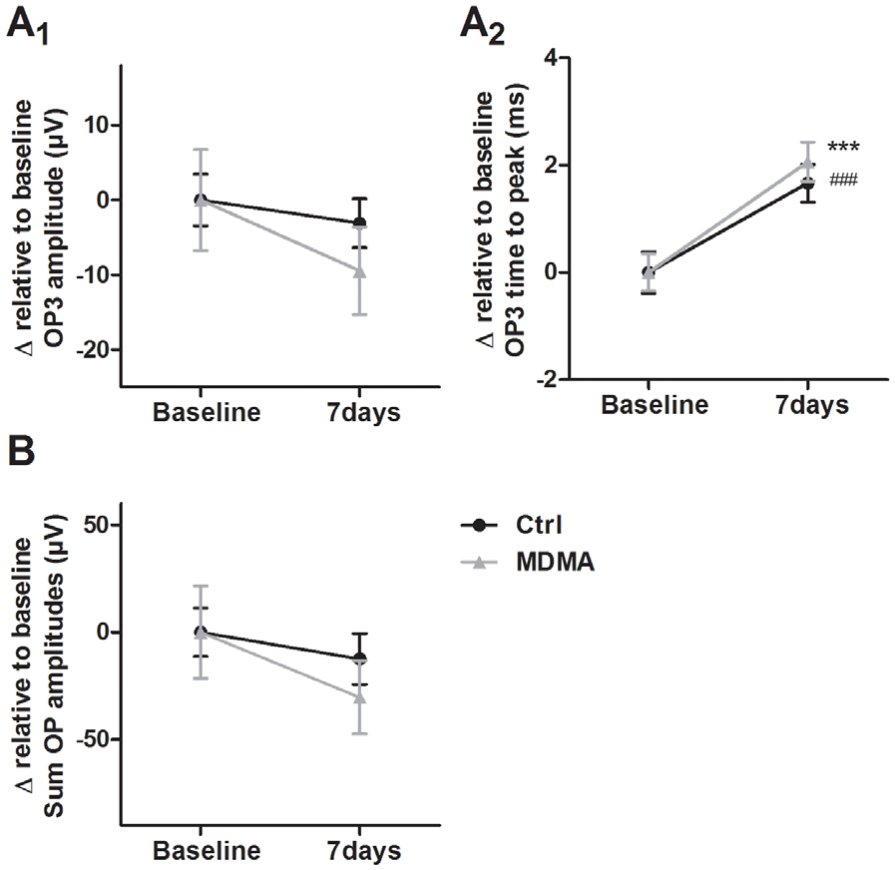
Effect of MDMA and HT on the scotopic OPs at 7 days. Four oscillatory potentials (OPs), OP1, OP2, OP3, and OP4, were extracted from scotopic ERGs. (A) Only the effect on the OP3 amplitude and time to peak is shown. (A_1_) No differences were found both in Control and MDMA groups at 7 days when compared to Baseline ERG. (A_2_) Time to peak values showed the same behavior for all OPs, in which both Control and MDMA groups presented increased time to peak values at 7 days when compared to Baseline ERG. (B) No differences were found both in Control and MDMA groups in the Sum of OP amplitudes at 7 days when compared to Baseline ERG. *** p<0.001 MDMA group compared to Baseline ERG; ### p<0.001 Control group compared to Baseline ERG. The data are presented as mean ± SEM.

Four oscillatory potential waveforms, OP1, OP2, OP3, and OP4, were extracted from scotopic ERGs elicited by five different light intensities (0.095 – 9.49 cd-s/m^2^). Concerning the 7 days time point, paired-samples *t* test showed that there were no significant differences in OP amplitudes (Fig 5A_1_) and in the Sum of OP amplitudes (Fig 5B) when compared to Baseline ERG. However, concerning time to peak values, there was a significant increase in both Control and MDMA groups at 7 days when compared to Baseline ERG (p<0.001, Fig 5A_2_). This result is in accordance with the increased time to peak values found in the b-wave (Fig 3B_2_) and suggests a slightly delayed response in both groups at 7 days when compared to Baseline ERG.

### Differential effects of MDMA and HT on ERG responses during photopic adaptation

**Figure 6:**
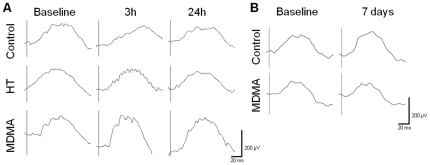
Effects of MDMA and HT on ERG during light adaptation. Example of individual ERGs recorded during light adaptation to a white background light (25 cd/m^2^), 24 h before saline or MDMA i.p. injection (Baseline ERG), and 3 h and 24 h after i.p. injection (A), and also recorded 7 days before (Baseline ERG) and 7 days after i.p. injection (B). ERGs were elicited by bright light flashes with an intensity of 9.49 cd-s/m^2^. Each trace represents an average of three responses. ERGs were recorded at the onset (0 min) and at 2, 4, 8, and 16 min of light adaptation. In this figure only the onset (0 min) is presented. Solid vertical line indicates the onset of flash light stimulus.

**Figure 7:**
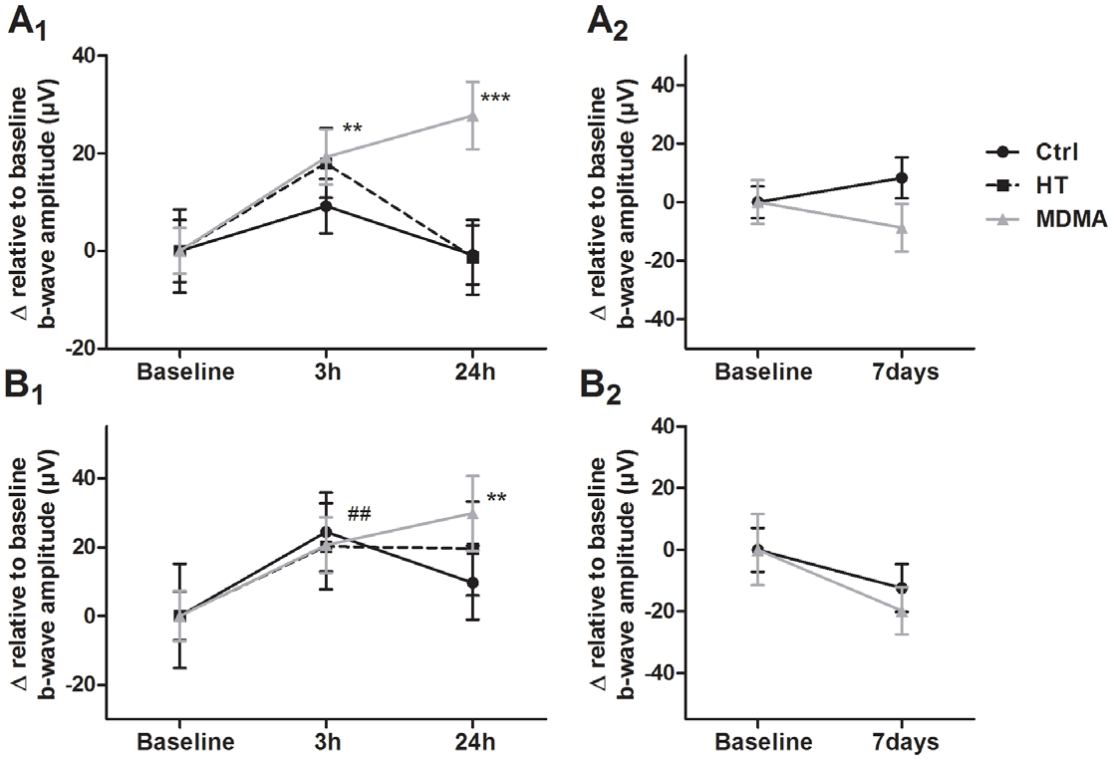
Effects of MDMA and HT on the photopic b-wave amplitude. (A) b-wave amplitude during light adaptation to a white background light (25 cd/m^2^). ERGs were recorded at the onset (0 min) and at 2, 4, 8, and 16 min of light adaptation. (B) Photopic b-wave (after light adaptation). ERGs were elicited by white bright flashes (3.00 and 9.49 cd-s/m^2^). (A_1_) Only the MDMA group had significantly increased b-wave amplitude during light adaptation at 3 h, when compared to Baseline ERG, though the HT group showed the same trend as the MDMA group (p = 0.044). At 24 h time point only the MDMA group presented increased b-wave amplitude when compared to Baseline ERG. (A_2_) At 7 days time point no differences were found in both Control and MDMA groups when compared to Baseline ERG. (B_1_) After light adaptation, increased photopic b-wave amplitude was found in Control group at 3 h when compared to Baseline ERG, though HT (p = 0,089) and MDMA (p = 0,017) groups may show similar behavior. At 24 h time point only MDMA group presented increased photopic b-wave amplitude when compared to Baseline ERG. (B_2_) No differences were found at 7 days time point when compared to Baseline ERG. ** p<0.01, *** p<0.001 MDMA compared to Baseline ERG; ## p<0.01 Control group compared to Baseline ERG. The data are presented as mean ± SEM.

ERGs were also recorded during light adaptation to a white background light (25 cd/m^2^), 24 h before saline or MDMA i.p. injection, and 3 h and 24 h after i.p. injection in the first study (Fig. 6A). In the second study, ERGs were recorded 7 days before i.p. injection (Baseline ERG) and 7 days after i.p. injection (Fig 6B). ERGs were elicited by bright light flashes with an intensity of 9.49 cd-s/m^2^ and were recorded at the onset (0 min) and at 2, 4, 8, and 16 min of light adaptation. For the b-wave amplitude values, the ANOVA repeated measures analysis indicated a main effect of time (F_(2,116)_ = 8.54; p<0.001) and a marginally significant interaction between time and group (p = 0.013) (Fig. 7A_1_). The post hoc analyses showed that only the MDMA group had increased b-wave amplitude values at 3 h (p<0.01) and 24 h (p<0.001). As the MDMA group, the HT group presented a similar trend to increase the b-wave amplitude at 3 h (p = 0.044), but not at 24 h time point, when compared to Baseline ERG (Fig 7A_1_). In the second study, however, the paired-samples *t* test found no significant differences in both Control and MDMA group at 7 days, when compared to Baseline ERG (Fig 7A_2_), suggesting that the increased b-wave amplitude induced by MDMA at 24 h is no longer present at 7 days. Regarding time to peak values, ANOVA repeated measures analysis indicated a main effect of time (F_(2,116)_ = 7.1; p<0.01) and a significant interaction between time and group (p<0.001). However, only the HT group reached statistically significant decreased values at 3 h time point when compared to Baseline ERG, while at 24 h time point there were no differences for all groups. Also, in the second study there were no significant differences in time to peak values at 7 days when compared to Baseline ERG (data not shown).

### MDMA effects on ERG responses following photopic adaptation

In the period that followed photopic adaptation, and regarding the b-wave amplitude, no significant interaction between time and group was found. Although at 24 h time point only the MDMA group presented a significant increase in the b-wave amplitude, when compared to Baseline ERG (p<0.01) (Fig 7B_1_), a similar trend was found in all groups at 3 h. However, the absence of an interaction effect in repeated measures analysis renders this effect questionable. In the second study, no significant differences were found in the b-wave amplitude in both Control and MDMA groups (Fig 7B_2_).

ANOVA repeated measures analysis indicated a main effect of time (F_(2,47)_ = 26.4; p<0.001) and a significant interaction between time and group for photopic b-wave time to peak values (p<0.001, data not shown). Data followed the same behavior of scotopic ERGs (paired *t* test analysis), in which both MDMA and HT groups presented decreased time to peak values at the 3 h time point (p<0.01 for MDMA, p<0.001 for HT), but no significant differences were found at 24 h time point when compared to Baseline ERG. This suggests that MDMA specific effects are not present. In the second study, no significant differences were found in time to peak values at 7 days when compared to Baseline ERG (data not shown).

A similar result to the b-wave amplitude was obtained for the photopic OP amplitudes in which no interaction between time and group was found. Nevertheless, in the OP time to peak values the ANOVA repeated measures analysis indicated a main effect of time for all OPs detected: F_(2,17)_ = 30.7; p<0.001 for OP2; F_(2,17)_ = 40; p<0.001 for OP3; and F_(2,17)_ = 18; p<0.001 for OP4; as well as a significant interaction between time and group (p<0.001 for the OP2, OP3, and OP4; concerning OP1 no waveform was detected). Post hoc paired *t* test analysis showed that both HT and MDMA groups had decreased time to peak values at 3 h time point when compared to Baseline ERG (for all OPs detected). In the second study, no significant differences were found in the OP amplitude and time to peak values in both Control and MDMA groups at 7 days when compared to Baseline ERG (data not shown).

Photopic flicker ERG results were analyzed using the Fast Fourier Transform (FFT). We considered the first two harmonics, which are the more representative of the original waveform. The corresponding values for the harmonic amplitude and phase correlate with ERG amplitude and time to peak, respectively. In the first study, the results from the photopic flicker test indicated that there was no significant interaction between time and group for the 1^st^ and 2^nd^ harmonic amplitude values of FFT. However, in the 1^st^ and 2^nd^ harmonic phase values of FFT, in a similar way to the time to peak values of scotopic and photopic ERG, the ANOVA repeated measures analysis indicated a main effect of time for the 1^st^ (F_(2,47)_ = 60.3; p<0.001) and the 2^nd^ (F_(2,47)_ = 58.1; p<0.001) harmonic components, as well as a significant interaction between time and group for the 1^st^ (p<0.001) and 2^nd^ (p<0.001) harmonic components. Moreover, post hoc paired *t* test analysis indicated that both MDMA and HT groups presented decreased phase values at 3 h time point (p<0.001), but no significant differences were found at 24 h time point when compared to Baseline ERG. In the second study no significant differences were observed in the photopic flicker ERG in both Control and MDMA groups at 7 days when compared to Baseline ERG (data not shown).

## Discussion

In this study, we have found direct evidence that MDMA causes acute and subacute enhancement of outer retinal responses. Since MDMA induces an acute increase in body temperature, light responses are consequently increased and accelerated. The increase in light responses may persist 24 h after drug administration. This effect was most prominent in the scotopic a- and b-waveforms (corresponding to photoreceptor/bipolar cell circuits), and could not be explained by temperature elevation (present only 3 h after treatment). Regarding photopic ERG, no interaction was found between time and group for some major parameters, namely the b-wave amplitude and photopic flicker. This result suggests that some MDMA specific effects are more subtle when the retina is light adapted. This also suggests that cone-based pathways are relatively less sensitive to subacute effects of MDMA than rod circuits. One week after MDMA administration the ERG alterations observed at 24 h were no longer present. No increased responses, either in the a-wave or in the b-wave, were found at 7 days. Also, in photopic responses, contrarily to the effect found at 24 h, no increased b-wave amplitude during light adaptation was observed 7 days after MDMA administration when compared to Baseline. Together, these results suggest that MDMA induces subacute ERG alterations, and a recovery occurs within one week of abstinence.

To our knowledge, this is the first study addressing the effects of MDMA administration on *in vivo* retinal neurophysiology, assessed by means of the rat ERG. The ERG changes found 3 h after MDMA administration can be mainly attributed to the increase in body temperature induced by MDMA, a well known effect of this drug, since the high temperature (HT) control animals had similar responses to those found in the MDMA group for the majority of the analyzed parameters, at this particular time point. These temperature-induced changes in ERG are consistent with previous studies in rat [31] and mouse [30]. Since the elevation in body temperature increased a-wave amplitude and decreased a-wave time to peak, it is likely that these changes reflect alterations in the rod photoresponses that are known to be temperature-dependent being higher and “accelerated” (fast onset) at higher temperatures in mammals [32]. Similar temperature-induced changes are likely to occur also in cone photoreceptors [33]. The augmented and accelerated light responses, due to body temperature increase, are likely to acutely affect visual perception in humans after MDMA ingestion in nightclubs or “rave” parties.

At the 24 h time point, the body temperature in animals from MDMA and HT groups was similar to Control group. However, only the MDMA group presented significantly increased a-wave amplitude, when compared to Baseline, suggesting the presence of a genuine pharmacological effect. It is important to note that the effect in the b-wave amplitude is likely to reside in the a-wave generating mechanisms, the photoreceptors, since no significant differences were found in the b/a ratio at both time points (no evidence for effect amplification). However, we cannot exclude the possibility that post-photoreceptor components are involved, since the a-wave peak amplitude has also been shown to reflect post-photoreceptor contributions [34].

Regarding the increased a-wave and b-wave amplitudes found 24 h after MDMA administration, we can attribute this augmented light response to a direct effect of MDMA since HT group show recovery from the values obtained at 3 h time point. For the OPs we cannot discard a contribution of temperature elevation in MDMA group since HT group also presented increased amplitudes.

MDMA administration induces acute and long-term disruption of the serotonergic system, although the dopaminergic system can be acutely affected as well [1], [2]. Moreover, MDMA has been shown to influence suprachiasmatic nucleus (SCN) circadian rhythm [35]. Taking these evidences into account, we suggest that the subacute alterations found in our recordings could be the result of MDMA-induced effects directly on the retinal serotonergic and/or dopaminergic system, or indirectly on SCN circadian rhythm although this explanation remains a speculation at this point.

In the mammalian retina, there is evidence for the presence of a mechanism for active 5-HT uptake [36] and various 5-HT receptors were identified [37]. In addition, 5-HT administered directly in the vitreous of cat eye increased b-wave amplitude [38]. The authors suggested that perhaps increased 5-HT uptake into GABAergic amacrine cells changes the functional properties of these cells therefore reducing GABAergic inhibition in the retina, and consequently increasing the light responses. Conversely, the destruction of 5-HT accumulating amacrine cells in the rabbit retina results in decreased b-wave amplitude, but increased OPs [39], which is consistent with the fact that OPs have a distinct origin from the b-wave and mainly originate from the inner retina in relation to oscillatory patterning in ganglion cells [40], [41]. However, these studies did not address whether these findings were directly due to the lack of 5-HT.

It has been shown that MDMA can alter the ability of SCN to phase shift in response to a photic or non-photic stimulus [35]. In addition, single or repeated administration of MDMA to rats phase shifts the circadian patterns of locomotor activity and wakefulness [42], [43]. Therefore, we hypothesize that in the present study MDMA administration may induce changes in serotonergic SCN or raphe nerve fibres, which have been shown to influence retinal physiology, including light responses [44], thus causing the subacutely altered ERGs observed. Furthermore, an altered SCN circadian pattern can influence the dopamine-melatonin cycle in the retina, which in turn, can greatly affect ERG responses [45], [46]. Indeed, DA levels in the retina are under circadian control, peaking during the day, which means that although more DA is released upon light stimulation, retinal DA-mediated effects occur even in the absence of light stimulation or light adaptation [47]. Based on these properties of DA, we hypothesize that even a slight shift in DA circadian phase could be responsible for the increased a-wave, due to strongly augmented rod photic responses, observed specifically in the MDMA group at the 24h time point.

In a recent study, Vielma and colleagues found increased a-wave, b-wave, and also OP amplitudes in rat eyes treated intravitreally with various nitric oxide (NO) donors [48]. The effect of NO was found under both scotopic and photopic conditions. The authors suggested that the NO-induced increase in S-nitrosation at photoreceptor level is responsible for ERG signal amplification since cGMP or cAMP pathways were not involved. Although previous studies reported reduced ERG amplitudes and retinal citotoxicity induced by NO donors [49], [50], in this study the authors showed that the effect of NO is dose-dependent and indeed, for higher doses, it decreases ERGs. MDMA administration was found to be associated with increased NOS activity and NO production [51]. Moreover, nitrosative stress has been associated with MDMA-induced 5-HT depletion [52]. Miranda and colleagues (2007) were able to reduce the MDMA toxic effects in the rat retina using an antioxidant with efficient NO and peroxynitrite scavenging capacity, CR-6 (3,4-dihydro-6-hydroxy-7-methoxy-2,2-dimethyl-1(2H)-benzopyran) [53]. Taking this into account, we can also speculate about the presence of elevated NO levels or S-nitrosation in the retina 24 h after MDMA administration. In this view, NO could also be involved in the increased amplitudes found in MDMA-treated animals at 3 h and 24 h.

To summarize, it was demonstrated for the first time that a single MDMA peripheral administration to rats induces an acute and subacute enhancement of outer retinal responses, as demonstrated by ERG recordings, which resume within one week of abstinence. Acute effects appear to be mostly due to the increase in body temperature (which is a well established acute effect of MDMA) since similar alterations were also detected in HT animals. Both MDMA and HT groups presented similar body temperature values (≈37.5°C) at this time point. Importantly, we also detected subacute (at 24 h time point) changes in the ERGs induced by MDMA administration. At this time point the animal body temperature was similar in all groups. Since the amplitude values for the a-wave were found to be significantly increased only in MDMA-treated animals, MDMA might induce higher rod photoreceptor light response. However, subacute increased light responses induced by MDMA, after single administration, were no longer present when ERGs were recorded after one week of MDMA abstinence. Therefore, a single administration of MDMA might induce temporary changes on retinal physiology, which may contribute to altered visual perception and visual acuity after MDMA use [17], [18], [19]. Human MDMA users may experience acute augmented light sensitivity due to hyperthermia, and some visual alterations might persist in the following day.

Future studies should address whether these changes in light responses persist after repeated MDMA administrations. Also, it will be important to evaluate the levels of retinal NO, 5-HT, and DA, after MDMA administration, in order to determine the significance of possible changes in this signaling molecules to MDMA-induced effects in light responses.
